# Gender Differences in the Relationships between Perceived Stress, Eating Behaviors, Sleep, Dietary Risk, and Body Mass Index

**DOI:** 10.3390/nu14051045

**Published:** 2022-02-28

**Authors:** Chen Du, Mary Adjepong, Megan Chong Hueh Zan, Min Jung Cho, Jenifer I. Fenton, Pao Ying Hsiao, Laura Keaver, Heesoon Lee, Mary-Jon Ludy, Wan Shen, Winnie Chee Siew Swee, Jyothi Thrivikraman, Felicity Amoah-Agyei, Emilie de Kanter, Wenyan Wang, Robin M. Tucker

**Affiliations:** 1 Department of Food Science and Human Nutrition, Michigan State University, East Lansing, MI 48824, USA; duchen@msu.edu (C.D.); imigjeni@msu.edu (J.I.F.); wangwe60@msu.edu (W.W.); 2 Department of Biochemistry, Kwame Nkrumah University of Science and Technology, Kumasi 00233, Ghana; madjepong2020@gmail.com (M.A.); felicityamoahagyei@gmail.com (F.A.-A.); 3 Division of Nutrition and Dietetics, International Medical University, Kuala Lumpur 57000, Malaysia; megan_chong@imu.edu.my (M.C.H.Z.); winnie_chee@imu.edu.my (W.C.S.S.); 4 Global Public Health, Leiden University College, 2595 DG The Hague, The Netherlands; m.j.cho@luc.leidenuniv.nl (M.J.C.); j.k.thrivikraman@luc.leidenuniv.nl (J.T.); emdekanter@gmail.com (E.d.K.); 5 Department of Food and Nutrition, Indiana University of Pennsylvania, Indiana, PA 15705, USA; pyhsiao@iup.edu; 6 Department of Health and Nutritional Science, Institute of Technology Sligo, F91 YW50 Sligo, Ireland; keaver.laura@itsligo.ie; 7 Department of Human Services, Bowling Green State University, Bowling Green, OH 43403, USA; leeh@bgsu.edu; 8 Department of Public and Allied Health, Bowling Green State University, Bowling Green, OH 43403, USA; mludy@bgsu.edu (M.-J.L.); wanshen@bgsu.edu (W.S.)

**Keywords:** restrained eating, uncontrolled eating, emotional eating, obesity, sleep, diet, college, COVID-19, international

## Abstract

Background: Obesity is a growing epidemic among university students, and the high levels of stress reported by this population could contribute to this issue. Singular relationships between perceived stress; engagement in restrained, uncontrolled, and emotional eating; sleep; dietary risk; and body mass index (BMI) have been reported in the current body of literature; however, these constructs interact with each other, and the complex relationships among them are infrequently examined. Therefore, the aim of the present study was to explore the complex relationships between these constructs using mediation and moderation analyses stratified by gender. Methods: A cross-sectional study, enrolling university students from the United States (U.S.), the Netherlands, South Korea, Malaysia, Ireland, Ghana, and China, was conducted between October 2020 and January 2021 during the COVID-19 pandemic. Perceived stress; maladaptive eating behaviors including restrained, uncontrolled, and emotional eating; sleep duration and quality; dietary risk; and BMI were assessed using validated questionnaires, which were distributed through an online platform. Results: A total of 1392 students completed the online survey (379 male, 973 female, and 40 who self-identified as “other”). Uncontrolled and emotional eating mediated the relationship between perceived stress and dietary risk for both males and females; higher sleep quality weakened this relationship among female students but not males. Emotional eating mediated the relationship between perceived stress and BMI for both males and females, but higher sleep quality weakened this relationship only among females. Conclusions: Our findings suggest that students in higher education are likely to benefit from interventions to reduce uncontrolled and emotional eating. Programs that improve sleep quality, especially during highly stressful periods, may be helpful.

## 1. Introduction

One in four university students are either overweight or obese worldwide [1]. Factors associated with excess adiposity among university students include, but are not limited to, dietary behaviors, poorer mental health [2], and high levels of stress [3,4,5]. These weight-gain promoting factors often co-exist [6,7,8]; for example, higher stress is associated with less healthy eating behaviors, such as higher energy intake [9], higher fat intake [10,11], more frequent consumption of sweets [12], and lower fruit and vegetable intake [13]. Conversely, an interventional study demonstrated a temporal relationship between stress and dietary choices, where decreased perceived stress through a stress management intervention reduced sweet snack intake [14]. Due to the complex nature of the relationships between stress and dietary behaviors, examining singular relationships between one independent and dependent variable can lead to an overly simplistic understanding of how these variables interact.

Further adding to the complexity of the relationships between weight status, dietary intake, and stress, a number of cognitive factors are also known to contribute to weight gain, and these can also be influenced by stress [15,16,17,18]. Three of these factors, restrained, uncontrolled, and emotional eating [19], are measured by the Three Factor Eating Questionnaire (TFEQ) [20]. These factors are also referred to as maladaptive eating behaviors [20]. Restrained eating refers to consciously avoiding or limiting the intake of certain foods [21,22,23], often with a goal to reduce body weight [24]. Restrained eating often leads to binge eating [25], as the act of dieting makes individuals vulnerable to disinhibition, which can result in overeating [23]. Uncontrolled eating can be described as a loss of control when eating certain foods [26], whereas emotional eating often refers to an over-consumption of food while experiencing negative emotions [27]. Females are reported to be more vulnerable to restrained, uncontrolled, and emotional eating behaviors [28,29,30,31]. These behaviors increase the risk of engaging in eating unhealthy foods, defined in this study and others as dietary risk [32,33], and promote weight gain [32,34]. Numerous studies indicate that individuals with restrained, uncontrolled, and emotional eating tend to overeat foods that are not consistent with health [35,36,37]. Additionally, individuals who frequently engage in restrained, uncontrolled, and emotional eating have a higher body mass index (BMI) compared to those who do not or who have low engagement with these behaviors [32,34,38]. Thus, the literature suggests temporal relationships between maladaptive eating behaviors, dietary risk, and BMI. More specifically, restrained, uncontrolled, and emotional eating can negatively influence dietary risk and weight status.

As stated above, perceived stress, described as the subjective perception of stress [39], is known to influence restrained, uncontrolled, and emotional eating behaviors [15,18,40]. Increases in perceived stress can induce restrained eating [40]. Stress can lead to a loss of control when eating, especially when eating hyperpalatable foods such as high-fat and sugary foods [18]. Additionally, higher perceived stress is associated with emotional eating [41], and the interaction between stress and emotional eating has a negative effect on BMI [15,42]. Taken together, this evidence suggests that higher levels of perceived stress could increase the frequency of engaging in maladaptive eating behaviors, including emotional, uncontrolled, and restrained eating. Given the temporal relationship between perceived stress and maladaptive eating behaviors, and the causal relationships between these behaviors and dietary risk and weight status [32,34,36], these maladaptive eating behaviors could mediate the relationships between perceived stress, dietary risk, and BMI.

Higher quality and adequate sleep are associated with lower stress [43,44], less frequent engagement in maladaptive eating behaviors identified by the TFEQ [45,46], lower dietary risk [47,48,49], and lower BMI [50,51]; therefore, sleep quality and duration could potentially moderate the relationships between these factors. Relationships between stress and sleep are bidirectional; that is, high levels of stress lead to shortened and poor sleep [52,53], and inadequate and poor sleep can exacerbate stress [54]. In terms of the relationships between stress and eating behaviors, previous work indicated that poor sleep quality is associated with increased frequency in emotional eating, and short sleep (<7 h per night) is associated with higher energy intake in emotional eaters [45]. Additionally, poor sleep quality and short sleep duration may induce stress and further exacerbate these maladaptive eating behaviors [45,46]. Moreover, the current body of literature has established associations between poor and inadequate sleep and higher BMI [50,51]. Given the evidence demonstrating the associations between sleep and stress, eating behaviors, dietary risks, and BMI, sleep could serve as a moderator of these relationships.

Previous studies examined the relationships between perceived stress, eating behaviors, dietary risk, BMI, and sleep in singular relationships; however, these factors interact with each other and should be examined in a more comprehensive manner. Therefore, the aim of the present study was to characterize the complex relationships between these factors using mediation and moderation analysis. Males and females appear to experience differences in terms of the degree and frequency of restrained, uncontrolled, and emotional eating [28,29,30,31]; thus, they were analyzed separately. Our hypotheses were: (1) maladaptive eating behaviors, including restrained eating, uncontrolled eating, and emotional eating, would mediate the relationships between perceived stress and dietary risk and perceived stress and BMI, and that the mediators could differ by gender; and (2) sleep quality and duration would moderate the relationship between perceived stress and dietary risk and perceived stress and BMI, and that the degree of moderation could differ by gender.

## 2. Materials and Methods

### 2.1. Study Design

Undergraduate and graduate students from universities in the United States (U.S.), the Netherlands, South Korea, Malaysia, Ireland, Ghana, and China were recruited. Only students who were at least 18 years old were included in the study. The study took place in late October 2020 to January 2021. Electronic surveys were used to collect information (see below), and were distributed via online platforms. All of the surveys were administered in English. During the data collection, Malaysia, South Korea, the Netherlands, and the U.S. had shelter-in-place orders, but China, Ghana, and Ireland did not. The study was approved by the Michigan State University Human Research Protection Program (East Lansing, MI, USA), STUDY00004285, 7 April 2020; Indiana University of Pennsylvania Institutional Review Board for the Protection of Human Subjects (Indiana, PA, USA), IRB Log 20-101, 26 October 2020; Bowling Green State University Office of Research Compliance (Bowling Green, OH, USA), 1,599,753 (US students), 29 April 2020; 1,599,753 (Chinese students), 22 May 2020; 1,599,753 (Korean students), 11 May 2020; Faculty of Governance and Global Affairs Ethics Committee (The Hague, South Holland, Netherlands), 2020-009-LUC-Cho, 25 May 2020; International Medical University Joint Committee on Research and Ethics (Kuala Lumpur, Malaysia), 481/2020, 14 May 2020; Institute Research Ethics Committee, Institute of Technology, Sligo (Sligo, Ireland), ref 2020015, 7 October 2020; and Kwame Nkrumah University of Science and Technology Committee on Human Research Publication and Ethics (School of Medical Sciences), CHRPE/AP/389/20, 30 October 2020. All participants consented to the study before filling out the online questionnaire.

### 2.2. Demographics and Biological Information

Age, gender, citizenship status (international vs. domestic), class status (undergraduate vs. graduate), and weight and height were all self-reported. Students who self-identified as neither male nor female were grouped as “other”, which included choices of transgender, genderqueer, additional gender category/other, or choose not to disclose. Race was collected only in the U.S. and Malaysia, and ethnicity was only collected in the U.S., as race and ethnicity diversification is more prevalent in these countries. Graduate status was defined as students who were pursuing masters, doctoral, or professional degrees; this information was collected because differences in stress [55], dietary risks [56], and BMI [57] between undergraduate and graduate students have been reported. International status was determined when students were attending universities outside of their home country. International status was collected as a covariate because it has been associated with the exposure (stress) and outcome variables (dietary risk and BMI) examined [58]. Self-reported weight and height were used to calculate body mass index (BMI).

### 2.3. Evaluation of Perceived Stress

The Perceived Stress Scale 10 (PSS-10) was used to assess perceived stress [39]. The questionnaire includes 10 questions, with four responses for each question ranging from 0 (never) to 4 (very often). One example question is, “In the last month, how often have you felt nervous and stressed?” The higher the total score, the more perceived stress there is. The highest possible PSS-10 score is 40 while the lowest is 0 [39].

### 2.4. Evaluation of Dietary Risk

The Starting the Conversation (STC) questionnaire was used to evaluate dietary risk [59]. This survey is a simplified food frequency questionnaire. The STC includes 8 questions that measure the consumption frequency of healthy foods (fruit, vegetables, and high-quality proteins) and unhealthy foods (chips, crackers, fast foods, desserts, solid fats, soda and sweet tea). One sample question is, “How many times a week did you eat regular snack chips or crackers (not low-fat)?” The possible answers include 1 time or less (0), 2 to 3 times (1), and 4 or more times (2). A global score ranging from 0 to 16 can be generated by the STC. A higher score means a higher frequency of engaging in unhealthy dietary behaviors [59].

### 2.5. Evaluation of Dietary Behaviors

The Three Factor Eating Questionnaire R18 (TFEQ-R18) was employed to examine the dietary behaviors of cognitive restraint, uncontrolled eating, and emotional eating [20]. The TFEQ-R18 includes 18 questions that measure maladaptive eating behaviors, and the instrument has been validated in many countries [60,61]. One example question is, “When I feel blue, I often overeat”. The possible answers include: “definitely true” (4), “mostly true” (3), “mostly false” (2), and “definitely false” (1). The TFEQ-R18 provides three subscale scores for the measured dietary behaviors. The formula, [(raw score-lowest possible raw score)/possible raw score range × 100], was used to transform subscale scores into scores on a scale of 0 to 100 [20]. Higher subscale scores indicate higher frequency of engaging in restrained eating caused by cognitive restraint, uncontrolled eating, or emotional eating.

### 2.6. Evaluation of Sleep Quality and Duration

The Pittsburgh Sleep Quality Index (PSQI) was used to determine subjective sleep quality [62,63]. The PSQI includes 10 questions assessing sleeping habits, duration, and degree of sleep problems, with scores ranging from 0 to 21. Higher PSQI scores indicate poorer sleep quality. Further to this, a PSQI score of ≥5 represents poor sleep quality while a PSQI score of <5 indicates good sleep quality [62].

Sleep duration was evaluated by asking participants the numbers of hours and minutes that they usually sleep for during weekdays and the weekend. A weighted method was used to calculate the average sleep duration of weekdays and weekends. The formula used was (((weekday sleep duration × 5) + (weekend sleep duration × 2))/7). Adequate sleep was defined by reporting an average sleep duration ≥7 h, while short sleep duration was defined as reporting average sleep duration <7 h [64].

### 2.7. Changes of Perceived Stress, Diet, and Sleep Due to the COVID-19 Pandemic

As the survey was conducted during the COVID-19 pandemic, changes in perceived stress, diet, and sleep due to the pandemic were assessed. Participants were asked whether and how their perceived stress, diet, and sleep quality and duration had changed during the COVID-19 pandemic compared to before the pandemic. For example, students were asked, “Have you made changes to your diet during the COVID-19 pandemic compared to before the pandemic?” The possible answers included eating healthier than before, eating less healthy than before, or no change.

### 2.8. Statistical Analysis

IBM SPSS Version 26 (IBM Corporation, Armonk, NY, USA) was used to analyze all data. Descriptive statistics are presented using percentages (%) or means ± standard deviations (SD). Outliers defined by greater or lower than mean ± 3SD were removed based on best practices for cleaning biological data such as BMI [65], and the variables examined were approximately normally distributed after outlier removal. Normal distribution was confirmed by skewness and kurtosis tests. To examine the relationships between the variables and covariates included in the mediation and moderation models, zero-order correlations were conducted. Significance was determined at *p* < 0.05. False discovery rate adjustment was performed to reduce the chance of type I error where appropriate, and the cut-off point was set at *q* = 0.05.

One-way analysis of variance (ANOVA) was conducted to determine gender differences in perceived stress, dietary risk, cognitive restraint, emotional eating, uncontrolled eating, sleep quality, and sleep duration. Bonferroni post hoc tests were performed, and significance was determined at *p* < 0.05. Students not identifying as either male or female were not included in the analysis due to the small sample size (*n* = 40).

**Figure 1:**
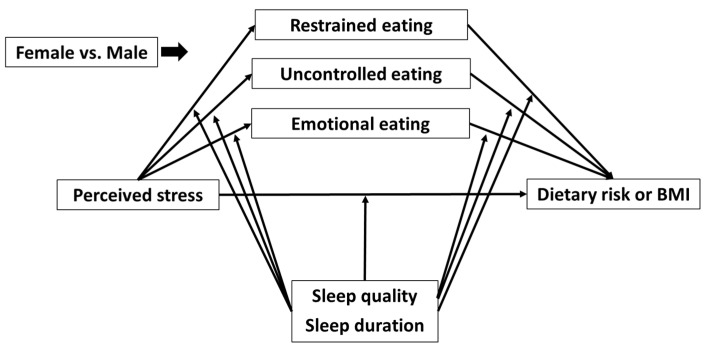
Model 1: The mediation effect of restrained, uncontrolled, and emotional eating on the relationship between perceived stress and dietary risk under the moderation of sleep quality for female students; Model 2: The mediation effect of restrained, uncontrolled, and emotional eating on the relationship between perceived stress and dietary risk under the moderation of sleep quality for male students; Model 3: The mediation effect of restrained, uncontrolled, and emotional eating on the relationship between perceived stress and dietary risk under the moderation of sleep duration for female students; and Model 4: The mediation effect of restrained, uncontrolled, and emotional eating on the relationship between perceived stress and dietary risk under the moderation of sleep duration for male students. Models A to D: same as Models 1 to 4 but BMI was the dependent variable. Note that “others” were excluded from analysis.

The SPSS PROCESS Macro was employed to conduct the moderated mediation analyses [66]. Model 59 was used. Previous studies suggest that testing mediation hypotheses using cross-sectional data is reasonable when temporal relationships between examined variables are shown [67,68,69]. Mediation analysis was used on the present cross-sectional dataset because temporal relationships between an independent variable (perceived stress), mediators (maladaptive eating behaviors), and dependent variables (dietary risk and BMI) are well established in the literature [15,18,23,26,40,70,71]. For example, increased stress leads to more frequent engagement in maladaptive eating behaviors [15,18,40], and these behaviors can further lead to the increased consumption of unhealthy foods and, thereafter, increased body weight [23,26,70,71]. Gender-specific models were developed because the mediation effects of cognitive constraint, uncontrolled eating, and emotional eating on the relationship between perceived stress and dietary risk have been shown to differ between males and females [28,29,30,31]. Students not identifying as either male or female were not included in the models because the sample size was too small (*n* = 40). A total of four models were built based on perceived stress as the independent variable and dietary risk as the dependent variable (Figure 1). The models were built to (1) examine the mediation effects of the three eating behaviors of interest on the relationship between perceived stress and dietary risk among female (Model 1) and male (Model 2) students, when under the moderation of sleep quality; and (2) examine the mediation effects of the three measured eating behaviors on the relationship between perceived stress and dietary risk among female (Model 3) and male (Model 4), when under the moderation of sleep duration. Four additional models were built based on perceived stress as the independent variable and BMI as the dependent variable. BMI was treated as a continuous variable. These models were similar to models 1 to 4, except BMI was the dependent variable (Model A—female students, sleep quality as the moderator; model B—male students, sleep quality as the moderator; Model C—female students, sleep duration as the moderator; Model D—male students, sleep duration as the moderator). Age, country, citizenship status, and class status were adjusted for all models, as these factors have been reported to be associated with either perceived stress, dietary risk, or BMI [57,72,73,74]. The number of bootstraps performed for bias corrected bootstrap confidence intervals was set at 10,000. *p* < 0.05 was used for determining significance for all analyses, and significant bootstrapping results were determined by the 95% confidence interval (CI) not crossing zero.

For interpreting the results of the mediation analysis, the following conditions were used [66]. (1) Significant bootstrapping results for indirect effect indicated a significant mediation effect of a mediator on the relationship between an independent and a dependent variable. (2) The significance of the mediation effect, also known as the indirect effect, does not depend on the singular relationship between an independent variable and a dependent variable (the direct effect), an independent variable and a mediator, and a mediator and a dependent variable being significant.

The following principles and conditions were used to interpret the results of the moderation analysis [66,75]. First, a significant association must be present between two variables in order for a moderator to moderate the relationship. Second, for a significant moderation effect, the Johnson-Neyman method can be used to identify a transition point. Third, both direct and indirect moderation relationships can be present. Direct moderation examines whether a moderator moderates the relationship between an independent and a dependent variable while holding mediators constant. On the other hand, indirect moderation examines two types of conditional relationships. First, an indirect moderation examines whether a moderator moderates the relationship between an independent variable and a mediator. Second, an indirect moderation examines whether a moderator also moderates the relationship between a mediator and a dependent variable. Examining the indirect effect of moderators takes changes in mediators into account, whereas examining direct effects does not. Therefore, examination of the indirect effects of the moderators was also conducted.

## 3. Results

### 3.1. Demographics and Variable Associations

A total of 1392 students completed the online survey (Table 1). The majority of the participants were female (70%), undergraduate (79%), and domestic students (82%).

The mean age of the students was 22 ± 5 years and the mean BMI was classified as healthy (Table 2). The average sleep quality was classified as poor, while the average sleep duration was adequate. Most of the students experienced increased perceived stress during the pandemic compared to before. More than one third of students reported less healthy eating, and nearly one third of students reported reduced sleep quality during the pandemic compared to before. Over 40% of students reported longer sleep duration during the pandemic compared to before the pandemic.

The zero-order correlations between variables tested and covariates revealed that perceived stress was positively associated with dietary risk, uncontrolled eating, emotional eating, sleep quality, and BMI, while negatively associated with sleep duration and age (Table 3). Sleep quality was measured using PSQI scores, where higher PSQI scores indicate poorer sleep quality. Additionally, dietary risk was positively associated with uncontrolled eating, emotional eating, sleep quality, and BMI, but negatively associated with cognitive restraint. Furthermore, uncontrolled eating was positively associated with emotional eating and sleep quality.

Sleep quality was negatively associated with sleep duration and positively associated with age and BMI. In addition, sleep duration was negatively associated with BMI. Age was negatively associated with BMI.

The one-way ANOVA analysis indicated that female students reported higher perceived stress and higher dietary risk (*p* < 0.001) compared to male students (Table 4). In terms of eating behaviors, female students reported higher restrained (*p* < 0.001) and emotional eating (*p* < 0.001) compared to male students. For sleep quality and duration, female students reported worse sleep quality (*p* = 0.003) but longer sleep duration compared to male students (*p* = 0.019).

### 3.2. Mediation and Moderation Analyses

#### 3.2.1. Model 1 Mediation of Eating Behaviors on the Relationship between Perceived Stress and Dietary Risk under the Moderation of Sleep Quality among Female Students

Model 1 mediation analysis revealed that perceived stress was positively associated with dietary risk among female students (Table 5). In addition, restrained eating negatively correlated with dietary risk, while uncontrolled and emotional eating positively correlated with dietary risk. Furthermore, uncontrolled (B = 0.0083, 95% CI = 0.0029, 0.0150) and emotional eating (B = 0.00138, 95% CI = 0.0068, 0.0223) significantly mediated the relationship between perceived stress and dietary risk among female students.

In order to examine whether sleep quality moderated the relationship between perceived stress and dietary risk directly or indirectly through the three eating behaviors (Table 6), two moderation pathways were evaluated. Sleep quality did not directly moderate the relationship between perceived stress and dietary risk, which was evidenced by the insignificant interaction effect of sleep quality and perceived stress on dietary risk (*p* = 0.6368) in Path 1 analysis. However, Path 2 analysis indicated that sleep quality moderated the relationship between perceived stress and dietary risk indirectly through moderating the relationship between uncontrolled eating and dietary risk and the relationship between emotional eating and dietary risk. These results were evidenced by the significant interaction effect of sleep quality and uncontrolled eating on dietary risk (*p* = 0.0388) and the significant interaction effect of sleep quality and emotional eating on dietary risk (*p* = 0.0223). Furthermore, based on the Johnson-Neyman test, higher sleep quality (lower PSQI scores) weakens the relationship between uncontrolled eating and dietary risk, and when the PSQI score is lower than 4.5 the relationship between uncontrolled eating and dietary risk disappears. Notably, 14.5% of female students reported a PSQI sore below 4.5, while 85.5% of female students reported a PSQI score above 4.5. Additionally, better sleep quality also weakens the relationship between emotional eating and dietary risk, and when the PSQI score is lower than 2.8, the relationship disappears.

Taken together, the mediation and moderation analyses of Model 1 indicated that the relationship between perceived stress and dietary risk was associated with uncontrolled eating and emotional eating among female students. Sleep quality moderated the relationship between perceived stress and dietary risk indirectly through uncontrolled and emotional eating.

#### 3.2.2. Model 2 Mediation of Eating Behaviors on the Relationship between Perceived Stress and Dietary Risk under the Moderation of Sleep Quality among Male Students

The Model 2 mediation analysis indicated that perceived stress positively correlated with uncontrolled eating (*p* = 0.0002) and emotional eating (*p* = 0.0021) among male students, while the other relationships presented in Table 7 were not significant, including a non-significant relationship between perceived stress and dietary risk. Additionally, restrained eating (B = −0.0076, 95% CI = −0.0189, 0.0018), uncontrolled eating (B = 0.0072, 95% CI = −0.0028, 0.0196), and emotional eating (B = 0.0057, 95% CI = −0.0046, 0.0178) did not mediate the relationship between perceived stress and dietary risk among male students.

To evaluate whether sleep quality moderated the relationship between perceived stress and dietary risk directly or indirectly through the measured eating behaviors among male students, two moderation pathways were assessed (Table 8). Path 1 analysis identified that sleep quality did not directly moderate the relationship between perceived stress and dietary risk, which was evidenced by the insignificant interaction effect of sleep quality and perceived stress on dietary risk (*p* = 0.1691). Additionally, Path 2 analysis indicated that sleep quality did not indirectly moderate the relationship between perceived stress and dietary risk, as the interaction effects of sleep quality and perceived stress on restrained eating (*p* = 0.4502), uncontrolled eating (*p* = 0.0740), and emotional eating (p = 0.2675) were insignificant, and the interaction effects of sleep quality and restrained eating (*p* = 0.5446), sleep quality and uncontrolled eating (*p* = 0.6480), and sleep quality and emotional eating (*p* = 0.2508) on dietary risk were insignificant.

In summary, these results confirmed that restrained, uncontrolled, and emotional eating did not mediate the relationship between perceived stress and dietary risk among male students, and that sleep quality did not moderate the relationship between perceived stress and dietary risk either directly or indirectly for male students.

#### 3.2.3. Model 3 Mediation of Eating Behaviors on the Relationship between Perceived Stress and Dietary Risk under the Moderation of Sleep Duration among Female Students

Model 3 mediation analysis reported that uncontrolled (B = 0.0041, 95% CI = 0.0022, 0.0109) and emotional eating (B = 0.0202, 95% CI = 0.0112, 0.0311) significantly mediated the relationship between perceived stress and dietary risk among female students, which agrees with the Model 1 mediation analysis. Unlike the mediation analyses, the Model 3 moderation analysis indicated that sleep duration did not directly or indirectly moderate the relationship between perceived stress and dietary risk among female students.

#### 3.2.4. Model 4 Mediation of Eating Behaviors on the Relationship between Perceived Stress and Dietary Risk under the Moderation of Sleep Duration among Male Students

The Model 4 mediation analysis indicated that restrained eating (B = −0.0053, 95% CI = −0.0171, 0.0064), uncontrolled eating (B = 0.0006, 95% CI = −0.0129, 0.0150), and emotional eating (B = 0.0157, 95% CI = −0.0002, 0.0370) did not mediate the relationship between perceived stress and dietary risk among male students. Furthermore, the Model 4 moderation analysis confirmed that sleep duration did not directly or indirectly moderate the relationship between perceived stress and dietary risk among male students.

#### 3.2.5. Model A Mediation of Eating Behaviors on the Relationship between Perceived Stress and BMI under the Moderation of Sleep Quality among Female Students

Model A mediation analysis revealed that emotional eating (B = 0.0244, 95% CI = 0.0116, 0.0406) significantly mediated the relationship between perceived stress and BMI among female students (Table 9). However, perceived stress was not significantly associated with restrained eating, uncontrolled eating, emotional eating, and BMI in this model. Only emotional eating was positively associated with BMI among female students (*p* = 0.0432).

To assess whether sleep quality moderated the relationship between perceived stress and BMI directly or indirectly through the three eating behaviors (Table 10), two moderation pathways were evaluated. Path 1 analysis indicated that sleep quality did not directly moderate the relationship between perceived stress and BMI, and this was shown by the insignificant interaction effect of sleep quality and perceived stress on BMI (*p* = 0.6710). However, Path 2 analysis revealed that sleep quality indirectly moderated the relationship between perceived stress and BMI through moderating the relationship between emotional eating and BMI. The result was evidenced by the significant interaction effect of sleep quality and emotional eating on BMI (*p* = 0.0154). Furthermore, based on the Johnson-Neyman test, better sleep quality weakens the relationship between emotional eating and BMI, and when the PSQI score is <4.7, the relationship between emotional eating and BMI disappears. Per the present data, 14.6% female students reported a PSQI score below 4.7, with the remainder of these students reporting a PSQI score above 4.7.

In summary, these results demonstrated that the relationship between perceived stress and BMI was associated with emotional eating among female students. Sleep quality moderated the relationship between perceived stress and BMI indirectly through emotional eating.

#### 3.2.6. Model B Mediation of Three Eating Behaviors on the Relationship between Perceived Stress and BMI under the Moderation of Sleep Quality among Male Students

Model B mediation analysis revealed that emotional eating (B = 0.0379, 95% CI = 0.0142, 0.0685) significantly mediated the relationship between perceived stress and BMI among male students. Additionally, perceived stress was positively associated with uncontrolled eating (*p* = 0.0001) and emotional eating (*p* = 0.0023) among male students.

Two moderation analyses were conducted in Model B. One pathway examined whether sleep quality moderated the relationship between perceived stress and BMI directly, and the other pathway tested whether sleep quality moderated the same relationship indirectly. The results indicated that sleep quality did not moderate the relationship between perceived stress and BMI directly or indirectly among male students. These results were supported by an insignificant interaction effect of sleep quality and perceived stress on BMI (*p* = 0.3526, direct effect) and the insignificant interaction effects of sleep quality and perceived stress on restrained eating (*p* = 0.6030), uncontrolled eating (*p* = 0.0550), and emotional eating (*p* = 0.2522). Additionally, the interaction effects of sleep quality and restrained eating (*p* = 0.8785), sleep quality and uncontrolled eating (*p* = 0.8333), and sleep quality and emotional eating on BMI were also insignificant (*p* = 0.9996).

Taken together, these results showed that the relationship between perceived stress and BMI was associated with emotional eating among male students. However, changes in sleep quality did not alter the relationship between perceived stress and BMI directly or indirectly among male students.

#### 3.2.7. Model C Mediation of Eating Behaviors on the Relationship between Perceived Stress and BMI under the Moderation of Sleep Duration among Female Students

Model C mediation analysis observed that emotional eating significantly mediated the relationship between perceived stress and BMI for females (B = 0.0506, 95% CI = 0.0167, 0.0959). The moderation analysis of Model C indicated that sleep duration did not directly or indirectly moderate the relationship between perceived stress and BMI.

#### 3.2.8. Model D Mediation of Eating Behaviors on the Relationship between Perceived Stress and BMI under the Moderation of Sleep Duration among Male Students

Model D mediation analysis indicated that emotional eating significantly mediated the relationship between perceived stress and BMI among male students (B = 0.0506, 95% CI = 0.0174, 0.0942). However, sleep duration did not directly or indirectly moderate the relationship between perceived stress and BMI.

## 4. Discussion

This study explored whether maladaptive eating behaviors, including restrained, uncontrolled, and emotional eating, mediated the relationships between perceived stress and dietary risk along with BMI, and further examined whether sleep quality and duration moderated these relationships. Results indicated that the relationship between perceived stress and BMI was mediated by emotional eating for both males and females; sleep quality, but not sleep duration, moderated this relationship indirectly through emotional eating, but only among females. Additionally, for female students, the relationship between perceived stress and dietary risk was mediated by uncontrolled eating and emotional eating, and sleep quality—but not duration—moderated this relationship indirectly, through uncontrolled and emotional eating. These findings suggest that reducing uncontrolled and emotional eating while improving sleep quality among female students, and reducing emotional eating among male students, could potentially reduce dietary risk and prevent weight gain among students experiencing high levels of stress.

### 4.1. The Mediation Effects of Retrained, Uncontrolled, and Emotional Eating on the Relationship between Perceived Stress and Dietary Risk

As hypothesized, study findings were influenced by gender. Uncontrolled eating and emotional eating mediated the relationship between perceived stress and dietary risk for females but not males. These observations are consistent with other reports where women tend to experience a higher frequency of uncontrolled and emotional eating compared to males [28,29,30,31]. Stress can trigger emotional eating [17], and stress-induced emotional eating is more common in women than men [17,35]. Furthermore, stress can lead to a greater tendency to overeat [1], especially among emotional eaters [76]. Both uncontrolled and emotional eating are characterized by eating a large quantity of foods that are highly palatable, such as sweets and high fat foods [18,27], which increase dietary risk. Thus, uncontrolled and emotional eating could serve as an intermediate connection between perceived stress and dietary risk, especially for female students.

Unlike what was hypothesized, restrained eating did not mediate the relationship between perceived stress and dietary risk, based on the insignificant bootstrapping result of the indirect effect of restrained eating on the relationship between perceived stress and dietary risk. Even though perceived stress is positively associated with dietary risk, and restrained eating is negatively associated with dietary risk, these singular relationships do not indicate a mediation effect [66]. A body of literature suggests that restrained eating is associated with overeating and higher dietary risk [77,78,79], and that restrained eaters are more likely to respond to stress by consuming highly palatable foods [80]. The discrepant results between this study and the work of others might be explained by the lack of relationship between perceived stress and restrained eating in our study sample. Students may tend to engage in other maladaptive eating behaviors such as emotional eating [81,82] during the COVID-19 pandemic. Little is known about the relationship between stress and restrained eating during the COVID-19 pandemic; however, one study reported that more severe COVID-19 lockdowns were associated with increased anxiety symptoms but lower restrained eating [83]. Thus, the stress related to the pandemic may help explain the lack of relationship between perceived stress and restrained eating in our study sample. In summary, restrained eating appears to have a limited association with dietary risk scores during the pandemic.

### 4.2. The Moderation Effects of Sleep Quality and Duration on the Relationship between Perceived Stress and Dietary Risk

As expected, sleep quality moderated the relationship between perceived stress and dietary risk indirectly by moderating the relationship between uncontrolled and emotional eating, but these relationships were only present in females. Furthermore, when the PSQI score was less than 4.5, the relationship between uncontrolled eating and dietary risk disappeared. To provide perspective, a PSQI score < 5 is considered to reflect good sleep quality. For emotional eating, when the PSQI score was less than 2.8, the relationship between emotional eating and dietary risk disappeared. Even though a PSQI score < 2.8 is clinically difficult to achieve, higher sleep quality weakening the effect of emotional eating on dietary risk is a practical finding. Therefore, improving sleep quality may weaken the relationship between perceived stress and dietary risk indirectly, by weakening the relationship between uncontrolled eating and dietary risk and by weakening the relationship between emotional eating and dietary risk for female students.

Unlike among female students, the same moderation effects of sleep quality on the relationship between perceived stress and dietary risk in males were not observed. The reason for this discrepancy is likely because uncontrolled and emotional eating did not mediate the relationship between perceived stress and dietary risk among these students. Furthermore, there were no associations among male students between either uncontrolled eating or emotional eating and dietary risk. Thus, improving sleep quality among male students may not reduce unhealthy dietary behaviors when under stress; however, better sleep quality is associated with better health outcomes later in life, such as lower cardiovascular disease [84,85,86] and infection risks [87,88] among both males and females. Therefore, even though a moderation effect of sleep quality on the relationship between perceived stress and dietary risk in males was not observed, good sleep quality is still important to the overall health of males.

### 4.3. The Mediation Effects of Retrained, Uncontrolled, and Emotional Eating on the Relationship between Perceived Stress and BMI

In the present study, results indicated that the relationship between perceived stress and BMI was associated with emotional eating for both males and females. These findings align with other studies where emotional eating mediated the relationship between negative emotion—including stress and depression—and weight gain in both males and females [34,70,71]. Furthermore, prospective studies conducted in Finland [34], France [89], South Korea [90], the Netherlands [71], and the U.S. [91] reported that higher levels of emotional eating were associated with greater weight gain. Additionally, stress tends to trigger undesirable food consumption among emotional eaters [17]. Therefore, targeting interventions that address emotional eating may help higher education students reduce risky dietary behaviors during stressful situations.

### 4.4. The Moderation Effects of Sleep Quality and Duration on the Relationship between Perceived Stress and BMI

Sleep quality moderated the relationship between perceived stress and BMI indirectly through emotional eating among female but not male students. This finding might be explained by female students suffering poorer sleep quality compared to male students. Several other studies reported similar findings where female young adults were more vulnerable to poor sleep quality [50,92,93]. Based on the literature and our findings, improved sleep quality may serve as an intervention target to disrupt the relationship between perceived stress and BMI indirectly, by weakening the relationship between emotional eating and BMI among female students.

Sleep duration did not moderate the relationship between perceived stress and dietary risk or BMI, which contradicts our hypothesis. These findings are inconsistent with studies that report that insufficient sleep leads to higher dietary risk and higher likelihood of weight gain [48,94,95,96], and that high stress can lead to shortened sleep [53,97]. The discrepancy between our findings and those reported by others might be explained by the fact that our study sample met recommended sleep duration guidelines, suggesting that many students achieved adequate sleep. Recent studies, along with our previous work, reported that students slept longer but with poorer sleep quality during the COVID-19 pandemic [98,99,100,101,102]. Additionally, over 40% of students in our study reported sleeping longer during the COVID-19 pandemic compared to before the pandemic. The current evidence points to focusing more on improving sleep quality rather than duration during a highly stressful event like the COVID-19 pandemic.

### 4.5. Public Health Messages

Our findings suggest that interventions focusing on helping students to address uncontrolled and emotional eating may be important strategies to reduce risky dietary behaviors and to prevent weight gain, especially during stressful times. However, screening for uncontrolled and emotional eating is not routinely conducted by universities [103,104]. Frequent engagement in uncontrolled and emotional eating increases the risk of developing eating disorders [105]. Eating disorders are more prevalent among university students compared to the general population [106,107]. Early detection of eating disorders and maladaptive eating behaviors is a key intervention for treating and preventing eating disorders [108]. Therefore, universities should consider implementing screenings for maladaptive eating behaviors for students and providing interventions as needed.

Improving sleep quality could serve as another target of intervention to help reduce dietary risk and prevent weight gain, especially among female students. However, there is currently a lack of programming for higher education institutions to address sleep issues [109,110]. Previous work reported that sleep disturbances are associated with academic success more strongly than drug use, drinking, and stress. However, screening for sleep issues and providing sleep hygiene interventions are not frequently conducted [111]. Besides screening, providing effective sleep programming also plays a key role in improving student sleep quality. Face-to-face [112,113] or virtual [114] delivery of cognitive behavior therapy for insomnia (CBTi) has been shown to be an effective strategy for improving sleep, and could help reduce dietary risk and prevent weight gain, especially during stressful situations.

### 4.6. Strengths and Limitations

The study has several strengths. In terms of study strengths, surveys were collected from a large population of students from seven countries, which broadens the generalizability of the results. Additionally, a more comprehensive examination of the relationships between perceived stress, eating behaviors, sleep, dietary risk, and BMI was performed using moderated mediation analyses. Furthermore, all instruments used in the study were validated, often in multiple countries [39,60,61,115]. Finally, the study was conducted during the COVID-19 pandemic, a time of heightened stress, which offers a unique perspective.

Several limitations are present in the study. First, the study is a cross-sectional study, in which the results only suggest relational, rather than causal, relationships between the variables examined. Therefore, a prospective longitudinal study is needed to confirm the relationship sequence suggested by the current study. Second, while the COVID-19 pandemic provided a unique time point to measure student stress, it also altered some student responses. A post-pandemic study is recommended to confirm the relationships observed. Third, the study did not control for the “shelter in place” status of each country included because the definition of “shelter in place” varied from country and country. Fourth, the study only included English speaking students, which excluded students who do not have English proficiency.

## 5. Conclusions

The current study demonstrated that uncontrolled and emotional eating mediated the relationship between perceived stress and dietary risk, and higher sleep quality weakened this relationship among female university students but not males. Additionally, emotional eating mediated the relationship between perceived stress and BMI for both males and females, but higher sleep quality weakened this relationship only among females. These findings suggest that interventions addressing uncontrolled and emotional eating, as well as sleep hygiene training, may be important strategies to reduce dietary risk and prevent weight gain among university students, especially when under considerable stress.

## Figures and Tables

**Table 1:** Demographics.

Location	Gender *n* (%)	Race	Ethnicity	Undergraduate vs. Graduates *n* (%)	Domestic vs. International *n* (%)
China	M = 26 (24) F = 77 (71) Other = 5 (5)	NA	NA	U = 3 (3) G = 104 (96)	D = 85 (79) I = 23 (21)
Ghana	M = 72 (56) F = 57 (44)	NA	NA	U = 113 (88) G = 16 (12)	D = 126 (98) I = 3 (2)
Ireland	M = 23 (15) F = 128 (84) Other = 2 (1)	NA	NA	U = 123 (80) G = 30 (20)	D = 133 (87) I = 20 (13)
Malaysia	M = 23 (23) F = 75 (74) Other = 3 (3)	Asian = 96 (95) Native Hawaiian or other Pacific Islander = 1 (1) Multiple races = 2 (2) Chose not to disclose = 2 (2)	NA	U = 67 (66) G = 34 (34)	D = 91 (90) I = 10 (10)
South Korea	M = 45 (43) F = 60 (57)	NA	NA	U = 78 (74) G = 27 (26)	D = 54 (51) I = 51 (49)
The Netherlands	M = 22 (23) F = 70 (75) Other = 2 (2)	NA	NA	U = 76 (81) G = 18 (19)	D = 37 (39) I = 57 (61)
United States	M = 168 (24) F = 506 (72) Other = 28 (4)	White = 548 (78) Black or African American = 14 (2) Asian = 86 (12) Other = 54 (8)	Hispanic = 37 (5) Non-Hispanic = 604 (86) Chose not to disclose = 61 (9)	U = 639 (91) G = 63 (9)	D = 618 (88) I = 84 (12)
Total	M = 379 (27) F = 973 (70) Other = 40 (3)	NA	NA	U = 1099 (79) G = 292 (21)	D = 1144 (82) I = 248 (18)

Note: M = male; F = female; Other = transgender, genderqueer, additional gender category (or other), and chose not to disclose; U = undergraduate students; G = graduate students; D = domestic students; I = international students.

**Table 2:** Demographics, health parameters, and changes in health parameters during the COVID-19 pandemic.

Demographics and Health Parameters	Mean (SD)
Age (years)	22.2 (5.4)
BMI (kg/m^2^)	23.9 (5.2)
Perceived stress (score)	21.2 (6.6)
Dietary risk (score)	7.8 (2.8)
Restrained eating (score)	31.8 (15.4)
Uncontrolled eating (score)	30.8 (15.3)
Emotional eating (score)	34.1 (22.6)
Sleep quality (score)	7.6 (3.2)
Sleep duration (h/night)	7.3 (1.2)
Changes in Health Parameters during the COVID-19 Pandemic	*n* (%)
Greater perceived stress	988 (71.0)
Eating less healthy	509 (36.6)
Reduced sleep quality	438 (31.5)
Reduced sleep duration	308 (22.1)
Increased sleep duration	558 (40.1)

Note: The PSS-10 was used to measure perceived stress, with total possible scores ranging from 0 to 40. A higher score indicates a greater amount of perceived stress. The STC questionnaire was used to measure dietary risk, and possible scores range from 0 to 16. Higher scores suggest greater risk. Restrained, uncontrolled, and emotional eating were measured using the TFEQ-R18. These measures can range from 0 to 100, with higher scores indicating more frequent engagement in these eating behaviors. The PSQI was used to measure sleep quality. The scores can range from 0 to 21. A PSQI score ≥ 5 indicates poor sleep quality; higher scores are undesirable. Sleep duration of ≥7 h per night is considered adequate as it meets minimum sleep duration recommendations.

**Table 3:** Zero order correlation of variables and covariates examined in the mediation/moderation models.

Measures	1	2	3	4	5	6	7	8	9
Perceived stress (1)	-	0.176 **	0.066 ^	0.189 **	0.275 **	0.412 **	−0.081 **	−0.097 **	0.055 *
Dietary risk (2)		-	0.228 **	0.188 **	0.251 **	0.219 **	−0.008	−0.034	0.154 **
Restrained eating (3)			-	0.003	0.061 ^	0.044	−0.046	0.049	0.054 ^
Uncontrolled eating (4)				-	0.640 **	0.183 **	−0.050	0.016	0.145 **
Emotional eating (5)					-	0.243 **	−0.026	0.036	0.231 **
Sleep quality (6)						-	−0.305 **	0.072 **	0.216 **
Sleep duration (7)							-	−0.028	−0.087 **
Age (8)								-	−0.178 **
BMI (9)									-

* *p* < 0.05. ** *p* < 0.01. ^ No longer significant after false discovery rate adjustment. The PSS-10 was employed to measure perceived stress; higher scores indicate greater perceived stress. Dietary risk was measured using the STC questionnaire, with higher scores indicating higher dietary risk. Restrained, uncontrolled, and emotional eating was measured using the TFEQ-R18, in which a higher score indicates more frequent engagement in these eating behaviors. Sleep quality was measured by PSQI scores. A higher PSQI score reflects poorer sleep quality.

**Table 4:** Gender differences in outcomes of interest.

	Perceived Stress	Dietary Risk	Restrained Eating	Uncontrolled Eating	Emotional Eating	Sleep Quality	Sleep Duration
Male	18.7 ± 6.8 ^a^	7.5 ± 2.8 ^a^	28.4 ± 14.0 ^a^	30.7 ± 15.0 ^a^	26.9 ± 21.7 ^a^	7.1 ± 2.9 ^a^	7.2 ± 1.1 ^a^
Female	22.0 ± 6.3 ^b^	8.0 ± 2.7 ^b^	33.2 ± 15.7 ^b^	30.9 ± 15.4 ^a^	36.8 ± 22.3 ^b^	7.7 ± 3.3 ^b^	7.4 ± 1.2 ^b^

Male: *n* = 379, female: *n* = 973; different superscripts in a column indicate significant differences. Note: Perceived stress was measured by the PSS-10, with scores ranging from 0 to 40; higher scores indicate greater perceived stress. Dietary risk was measured using the STC questionnaire, with higher scores indicating higher dietary risk. The scores range from 0 to 16. Restrained, uncontrolled, and emotional eating were measured using the TFEQ-R18, in which a higher score indicates more frequent engagement in these eating behaviors. These measures range from 0 to 100. Sleep quality was measured by PSQI scores, ranging from 0 to 21; higher scores indicate worse sleep quality.

**Table 5:** Model 1 mediation analysis of eating behaviors on the relationship between perceived stress and dietary risk for females.

Variables	B	SE	T	*p* Value
Perceived stress → restrained eating	0.0192	0.2260	0.0850	0.9322
Perceived stress → uncontrolled eating	0.3782	0.2116	1.7871	0.0742
Perceived stress → emotional eating	0.4428	0.3011	1.4703	0.1418
Perceived stress → dietary risk	0.0179	0.0353	0.5060	0.0131
Restrained eating → dietary risk	−0.0313	0.0147	−2.1259	0.0338
Uncontrolled eating → dietary risk	0.0266	0.0065	4.0853	<0.0001
Emotional eating → dietary risk	0.0240	0.0044	5.5011	<0.0001
Bootstrap	Effect	SE	LL95% CI	UL95% CI
Restrained eating	−0.0014	0.0039	−0.0092	0.0065
Uncontrolled eating	0.0083	0.0031	0.0029	0.0150
Emotional eating	0.00138	0.0040	0.0068	0.0223

Note: female students *n* = 964. B = beta coefficient; SE = standard error; T = *t*-test statistic. → indicates the relationship between the two variables.

**Table 6:** Model 1 moderation analysis for females (sleep quality as a moderator).

Variables	B	SE	T	*p* Value
Moderation pathway 1—direct effect of sleep quality on perceived stress and dietary risk				
Sleep quality × perceived stress → dietary risk	0.0021	0.0044	0.4723	0.6368
Moderation pathway 2—indirect effect of sleep quality on perceived stress and dietary risk				
Sleep quality → restrained eating	0.3046	0.6642	0.4586	0.6466
Sleep quality → uncontrolled eating	0.8758	0.6249	1.4026	0.1614
Sleep quality → emotional eating	0.4684	0.8605	0.5443	0.5864
Sleep quality × perceived stress → restrained eating	0.0021	0.0289	0.0723	0.9424
Sleep quality × perceived stress → uncontrolled eating	−0.0095	0.0271	−0.3506	0.7260
Sleep quality × perceived stress → emotional eating	0.0186	0.0367	0.5077	0.6118
Sleep quality × restrained eating → dietary risk	−0.0025	0.0018	−1.4253	0.1544
Sleep quality × uncontrolled eating → dietary risk	0.0034	0.0018	1.8215	0.0388
Sleep quality × emotional eating → dietary risk	0.0021	0.0012	1.7394	0.0223

Note: female students *n* = 964. B = beta coefficient; SE = standard error; T = *t*-test statistic. → indicates the relationship between the two variables.

**Table 7:** Model 2 mediation Analysis of eating behaviors on the relationship between perceived stress and dietary risk for males.

Variables	B	SE	T	*p* Value
Perceived stress → restrained eating	−0.0612	0.3544	−0.1726	0.8631
Perceived stress → uncontrolled eating	1.0656	0.2814	3.7871	0.0002
Perceived stress → emotional eating	1.2048	0.3889	3.0980	0.0021
Perceived stress → dietary risk	1.8040	1.0969	1.6447	0.1009
Restrained eating → dietary risk	−0.0291	0.0250	−1.1649	0.2448
Uncontrolled eating → dietary risk	0.0022	0.0278	0.0781	0.9378
Emotional eating → dietary risk	−0.0111	0.0183	−0.0636	0.5465
Bootstrap	Effect	SE	LL95% CI	UL95% CI
Restrained eating	−0.0076	0.0052	−0.0189	0.0018
Uncontrolled eating	0.0072	0.0056	−0.0028	0.0196
Emotional eating	0.0057	0.0056	−0.0046	0.0178

Note: male students *n* = 374. B = beta coefficient; SE = standard error; T = *t*-test statistic. → indicates the relationship between the two variables.

**Table 8:** Model 3 moderation analysis (sleep quality as a moderator).

Variables	B	SE	T	*p* Value
Moderation pathway 1—direct effect of sleep quality on perceived stress and dietary risk				
Sleep quality × perceived stress → dietary risk	−0.0084	0.0061	−1.3779	0.1691
Moderation pathway 2—indirect effect of sleep quality on perceived stress and dietary risk				
Sleep quality → restrained eating	−1.1201	0.9038	−1.2394	0.2160
Sleep quality → uncontrolled eating	1..6613	0.9169	1.8118	0.0708
Sleep quality → emotional eating	1.8040	1.0969	1.6447	0.1009
Sleep quality x perceived stress → restrained eating	0.0343	0.0453	0.7559	0.4502
Sleep quality × perceived stress → uncontrolled eating	−0.0756	0.0422	−1.7919	0.0740
Sleep quality × perceived stress → emotional eating	−0.0592	0.0534	−1.1104	0.2675
Sleep quality × restrained eating→ dietary risk	−0.0019	0.0032	−0.6065	0.5446
Sleep quality × uncontrolled eating → dietary risk	0.0016	0.0035	0.4569	0.6480
Sleep quality × emotional eating → dietary risk	0.0026	0.0023	1.1501	0.2508

Note: male students *n* = 374. B = beta coefficient; SE = standard error; T = *t*-test statistic. → indicates the relationship between the two variables.

**Table 9:** Model A mediation analysis of eating behaviors on the relationship between perceived stress and BMI for females.

Variables	B	SE	T	*p* Value
Perceived stress → restrained eating	0.0472	0.2349	0.2007	0.8410
Perceived stress → uncontrolled eating	0.4180	0.2186	1.9123	0.0562
Perceived stress → emotional eating	0.5217	0.3108	1.6783	0.0936
Perceived stress → BMI	−0.0616	0.0724	−0.8508	0.3951
Restrained eating → BMI	0.0415	0.0308	1.3488	0.1777
Uncontrolled eating → BMI	0.0510	0.0395	1.2926	0.1965
Emotional eating → BMI	0.0124	0.0233	0.5344	0.0432
Bootstrap	Effect	SE	LL95% CI	UL95% CI
Restrained eating	0.0005	0.0017	−0.0027	0.0042
Uncontrolled eating	0.0005	0.0046	−0.0086	0.0100
Emotional eating	0.0244	0.0076	0.0116	0.0406

Note: female students *n* = 964. B = beta coefficient; SE = standard error; T = *t*-test statistic. → indicates the relationship between the two variables.

**Table 10:** Model A moderation analysis (sleep quality as a moderator) for females.

Variables	B	SE	T	*p* Value
Moderation pathway 1—direct effect of sleep quality on perceived stress and BMI				
Sleep quality × perceived stress → BMI	0.0042	0.0099	0.4249	0.6710
Moderation pathway 2—indirect effect of sleep quality on perceived stress and BMI				
Sleep quality → restrained eating	0.4611	0.6836	0.6745	0.5002
Sleep quality → uncontrolled eating	0.8809	0.6409	1.3744	0.1696
Sleep quality → emotional eating	0.5190	0.8793	0.5902	0.5552
Sleep quality × perceived stress → restrained eating	−0.0018	0.0299	−0.0608	0.9515
Sleep quality × perceived stress → uncontrolled eating	−0.0127	0.0280	−0.4548	0.6494
Sleep quality × perceived stress → emotional eating	0.0128	0.0377	0.3382	0.7353
Sleep quality × restrained eating → BMI	−0.0040	0.0041	−0.9773	0.3287
Sleep quality × uncontrolled eating → BMI	−0.0070	0.0049	−1.4532	0.1465
Sleep quality × emotional eating → BMI	0.0075	0.0031	2.4279	0.0154

Note: female students *n* = 964. B = beta coefficient; SE = standard error; T = *t*-test statistic. → indicates the relationship between the two variables.

## Data Availability

The data presented in this study are available on request from the corresponding author. The data are not publicly available due to ongoing analyses.
